# Gut Microbiota in Patients with Prediabetes

**DOI:** 10.3390/nu16081105

**Published:** 2024-04-09

**Authors:** Wei-Lin Chang, Yu-En Chen, Hsiang-Tung Tseng, Ching-Feng Cheng, Jing-Hui Wu, Yi-Cheng Hou

**Affiliations:** 1 Department of Nutrition, Taipei Tzu-Chi Hospital, Buddhist Tzu-Chi Medical Foundation, New Taipei City 23142, Taiwan; mono5200@gmail.com (W.-L.C.); willa91308@gmail.com (Y.-E.C.); ma07110014@tmu.edu.tw (H.-T.T.); rita@tzuchi.com.tw (J.-H.W.); 2 Department of Pediatrics, Taipei Tzu-Chi Hospital, Buddhist Tzu-Chi Medical Foundation, New Taipei City 23142, Taiwan; chengcf@mail.tcu.edu.tw; 3 Institute of Biomedical Sciences, Academia Sinica, Taipei City 11529, Taiwan; 4 Department of Pediatrics, Tzu Chi University, Hualien 970374, Taiwan

**Keywords:** gut microbiota, prediabetes, high-fiber diet, high-carbohydrate diet, dietary control

## Abstract

Prediabetes is characterized by abnormal glycemic levels below the type 2 diabetes threshold, and effective control of blood glucose may prevent the progression to type 2 diabetes. While the association between the gut microbiota, glucose metabolism, and insulin resistance in diabetic patients has been established in previous studies, there is a lack of research regarding these aspects in prediabetic patients in Asia. We aim to investigate the composition of the gut microbiota in prediabetic patients and their differences compared to healthy individuals. In total, 57 prediabetic patients and 60 healthy adult individuals aged 18 to 65 years old were included in this study. Biochemistry data, fecal samples, and 3 days of food records were collected. Deoxyribonucleic acid extraction and next-generation sequencing via 16S ribosomal ribonucleic acid metagenomic sequencing were conducted to analyze the relationship between the gut microbiota and dietary habits. Prediabetic patients showed a lower microbial diversity than healthy individuals, with 9 bacterial genera being less abundant and 14 others more abundant. Prediabetic patients who consumed a low-carbohydrate (LC) diet exhibited higher diversity in the gut microbiota than those who consumed a high-carbohydrate diet. A higher abundance of *Coprococcus* was observed in the prediabetic patients on an LC diet. Compared to healthy individuals, the gut microbiota of prediabetic patients was significantly different, and adopting an LC diet with high dietary fiber consumption may positively impact the gut microbiota. Future studies should aim to understand the relationship between the gut microbiota and glycemic control in the Asian population.

## 1. Introduction

Prediabetes, defined by a fasting blood glucose level of 100 to <126 mg/dL or glycated hemoglobin of 5.7% to <6.5%, represents a high-risk state for developing diabetes. According to the American Diabetes Association, up to 70% of prediabetic cases eventually progress to diabetes, highlighting the urgency for effective intervention [1]. Beyond the immediate health concerns, prediabetes poses an increased risk of complications and mortality and has placed a significant burden on healthcare systems and national economies [2]. Available interventions, such as lifestyle changes [3], medication [4], and bariatric surgery [5], have been proven to be effective in preventing or delaying the progression of prediabetes to type 2 diabetes. However, a high proportion of prediabetic patients still develop diabetes with the existing intervention strategy, underlining the importance of developing interventions to prevent or even reverse the progression of prediabetes.

Previous studies have shown that the intestinal bacteria play a crucial role in the regulation of glucose and lipid metabolism, and altered composition of the intestinal bacteria has been linked to various diseases [6]. Changes in intestinal bacteria can compromise intestinal permeability, causing bacterial lipopolysaccharide to enter the bloodstream and induce insulin resistance, thereby increasing the risk of diabetes [7]. In addition, compromised gut barrier integrity has been observed in diabetic patients and has been identified both as the cause and the result of type 2 diabetes [8,9]. An altered gut microbe is directly correlated with the increased permeability of the gut, as intestinal bacteria interact with the epithelium of the gut to regulate the tight junctions of the epithelial structure [10]. Increased gut permeability promotes low-grade systemic inflammation, which is a root cause for metabolic disorders and various chronic diseases, including type 2 diabetes [8]. These findings are supported by recent studies that found markers such as zonulin and short-chain-fatty-acid-producing bacteria in the gut to have a correlation with diabetes and its complications [8,11]. Thus, investigating the relationship between intestinal bacteria alteration and a compromised gut barrier on the development of diabetes, and the potential factors causing said changes, is imperative to attenuate the progression of prediabetes to diabetes.

In an attempt to recover the altered gut microbiota present in various gastrointestinal diseases, a fecal microbiome transplant (FMT) is an option. The transfer of a small amount of feces from a healthy donor to a recipient has been found to correct the ratio of gut microbes and therefore attenuate the gastrointestinal and metabolic problems caused by gut dysbiosis [12,13,14,15]. A recent meta-analysis found that though in the short term (≤6 months) FMT improves fasting blood glucose, glycated hemoglobin, and plasma insulin levels, there are only slight long-term improvements (≥12 months) on glycated hemoglobin [15]. Diet alterations, on the other hand, have a long-term effect on the metabolic markers of diabetes [16].

Various factors, including diet [17], age [18], and the use of drugs such as antibiotics [19], may lead to alterations in the gut microbiota. Notably, diet has been identified as the primary factor exerting the most substantial impact on the human gut microbiota [20]. For example, fermentable dietary fiber serves as an energy source for beneficial intestinal bacteria, fostering the production of short-chain fatty acids and maintaining gut bacterial diversity [21]. Studies have shown that the abundance of butyrate-producing bacteria differs between healthy individuals and people with diabetes, suggesting that the gut microbiome may play a role in blood glucose regulation.

While the difference in intestinal bacteria between diabetic patients and healthy individuals is well established [22], information regarding intestinal bacteria composition and its impact on physiological mechanisms in prediabetic patients in Asian populations is still lacking [23]. This study aims to investigate the composition of the gut microbiota in prediabetic patients and their differences compared to healthy individuals. Additionally, the relationship between diet and the gut microbiota in prediabetic patients is further analyzed to provide insights that may contribute to the development of nutrition interventions in preventing the progression of prediabetes.

## 2. Methods

This study enrolled subjects with prediabetes from the Outpatient Department of Family Medicine, Taipei Tzu-Chi Hospital, New Taipei City, Taiwan (R. O. C.). Prediabetes was diagnosed based on a fasting blood glucose value of 100 to <126 mg/dL and a hemoglobin A1c of 5.7% to <6.5%. The inclusion criteria of the individuals included the following: (1) age range of 18–65 years old and (2) not having taken antibiotics, probiotics, or both within 8 weeks prior to fecal specimen collection. A total of 74 subjects with prediabetes were eligible for screening, and after selecting these individuals according to the inclusion criteria, 57 subjects with prediabetes were enrolled for this study. All individuals were provided with written informed consent on the day of recruitment. Data on the gut microbiota of 60 healthy individuals aged 18 to 65 years were obtained from a biobank [24]. The study protocol was approved by the institutional review board of the Taipei Tzu-Chi Hospital (Approval Code: 07-XD-061; Approval Date: 21 September 2018) and funding was provided under grant numbers TCRD-TPE-109-RT-10, TCRD-TPE-111-65, TCRD-TPE-112-RT-9, and TCRD-TPE-108-38. This study followed the STROBE statement and checklist for cross-sectional studies.

On the day of recruitment, subjects received instructions from research assistants to keep a 3-day food record and collect a fecal sample on the third day of the record. The subjects were told to record (1) the time they ate/drank during the day, (2) every food/drink/condiment consumed, and (3) the estimation of the food portion eaten using household utensils (e.g., bowls/cups/tablespoons). Record sheets with the written instructions mentioned above were provided to the subjects. The 3 days of food records consisted of 2 weekdays and 1 day on the weekend. Carbohydrate intake was defined as follows: high carbohydrate (HC), carbohydrate intake of ≥55% total calories; low carbohydrate (LC), carbohydrate intake of <55% total calories. Crude protein intake was defined as follows: high crude protein (HCP), crude protein intake of ≥16% total calories; low crude protein (LCP), crude protein intake of <16% total calories. Crude fat intake was defined as follows: high fat (HF), crude fat intake of ≥33% total calories; low fat (LF), crude fat intake of <33% total calories. Dietary fiber intake was defined as follows: high dietary fiber (HDF), dietary fiber intake of ≥14 g per day; low dietary fiber (LDF), dietary fiber intake of <14 g per day. Calorie intake was defined as follows: female high calorie (FHK), intake of ≥1500 kcal per day; female low calorie (FLK), intake of <1500 kcal per day; male high calorie (MHK), intake of ≥1800 kcal per day; male low calorie (MLK), intake of <1800 kcal per day.

The nutrient composition of their diet was calculated using Nutritionist Professional software 2.0 (E-Kitchen Business, Taichung, Taiwan). The software uses a nutrient database based on the Taiwan food composition table from the Ministry of Health and Welfare, Taiwan, ROC.

Fecal samples were collected using an LIBO feces collection container and transported to the laboratory at 4 °C. The samples were immediately centrifuged at 13,000× *g* for 1 min. After removing the supernatant, the samples were stored at −20 °C until deoxyribonucleic acid (DNA) extraction. The physical bead-beating method was applied to the samples to achieve better bacterial lysis before DNA was purified using a QIAamp PowerFecal DNA Kit (QIAGEN, Germantown, MD, USA).

16S ribosomal ribonucleic acid (rRNA) sequencing libraries were prepared according to the manufacturer’s instructions (Illumina, San Diego, CA, USA). Briefly, 12.5 ng of DNA was used for the polymerase chain reaction (PCR) amplification of the V3 and V4 regions of the 16S rRNA gene. The PCR primers contained an overhang adapter sequence, and the full-length primer sequences were as follows:

Forward: 5′-TCGTCGGCAGCGTCAGATGTGTATAAGAGACAGCCTACGGGNGGCWGCAG

Reverse: 5′-GTCTCGTGGGCTCGGAGATGTGTATAAGAGACAGGACTACHVGGGTATCTAATCC

The PCR products were purified using AMPure XP beads (Beckman Coulter, Brea, CA, USA) and subjected to a secondary PCR with primers from a Nextera XT Index kit (Illumina, USA) by adding dual indices and Illumina sequencing adapters. After PCR, the final libraries (~630 bp) were purified using AMPure XP beads and were ready for next-generation sequencing. The concentration of the 16S rRNA sequencing libraries was determined using real-time quantitative PCR with Illumina adapter-specific primers provided by a KAPA Library Quantification Kit (KAPA Biosystems, Wilmington, MA, USA). Libraries were denatured and sequenced using the Illumina MiSeq platform with Reagent v3 for paired-end sequencing (2 × 250 base pairs). Instrument control, cluster generation, image capture, and base calling were processed using real-time analysis software and MiSeq Control software on the MiSeq platform.

Whole-sequencing data will be made public and downloadable after 16 October 2026 from http://www.ncbi.nlm.nih.gov/bioproject/891488.

Sequencing reads were subjected to quality control using the FASTX-Toolkit (http://hannonlab.cshl.edu/fastx_toolkit/; accessed on 14 January 2022) with the following parameters: fastq_quality_filter -Q33 -q 20 -p 70; fastq_quality_trimmer -t 20 -l 200 -Q33. Quality control process reads were used to create a Zero-radius Operational Taxonomic Unit using the USEARCH UNOISE algorithm (https://drive5.com/usearch/manual/pipe_otus.html; accessed on 16 January 2022). Taxonomic identification was performed by aligning the Zero-radius Operational Taxonomic Unit with SILVA database 138 (https://www.arb-silva.de/; accessed on 14 January 2022). Basic Local Alignment Search Tool (BLAST) (https://blast.ncbi.nlm.nih.gov/Blast.cgi; accessed on 11 January 2022) was used as the alignment tool with a sequence similarity threshold of 97%. Principal coordinate analysis, alpha and beta diversity analyses, and visualization were performed using several R packages, such as vegan (https://cran.r-project.org/web/packages/vegan/index.html; accessed on 8 February 2022), PCAtools (https://bioconductor.org/packages/release/bioc/html/PCAtools.html; accessed on 1 March 2022), and ggbiplot (https://www.rdocumentation.org/packages/ggbiplot/versions/0.55; accessed on 8 February 2022). Linear discriminant analysis (LDA) and linear discriminant effect size (LEfSe) analysis were performed using the Lefse package (https://github.com/SegataLab/lefse; accessed on 8 February 2022). *p*-values were calculated using Kruskal–Wallis and Wilcoxon rank-sum tests. Significant bacterial genera were identified based on the following criteria: (a) *p* < 0.05, (b) fold change more than doubled, (c) average relative abundance more than 0.5% in at least one group, and (d) LDA score > 3. The pathway activity was calculated using the Tax4Fun package (http://tax4fun.gobics.de/; accessed on 17 February 2022). Furthermore, the *p*-value was calculated using the analysis of variance test.

## 3. Results

In total, 117 samples were collected for this study, consisting of 60 healthy individuals and 57 prediabetic individuals. The age of control group was significantly less than prediabetic group (52.6 ± 14.1 vs. 58.2 ± 6.0 years old; *p* = 0.006). Prediabetic group also had a higher BMI comparing to the controls (24.7 ± 3.8 vs. 22.9 ± 2.2 kg/m^2^; *p* = 0.003). The intakes of carbohydrates, proteins, and fat in a 24 h period by prediabetic patients are listed in Supplementary Table S1. Patients with prediabetes were split into five groups according to the nutrient intake ratio, defined based on the 2013–2016 Nutrition and Health Survey data in Taiwan (Table 1).

The data show that the intake of carbohydrates, protein, and fat in adults is 50–59%, 15–17%, and 24–32% kcal, respectively, and the intake of dietary fiber is 13.5–18.5 g/day.

**Figure 1:**
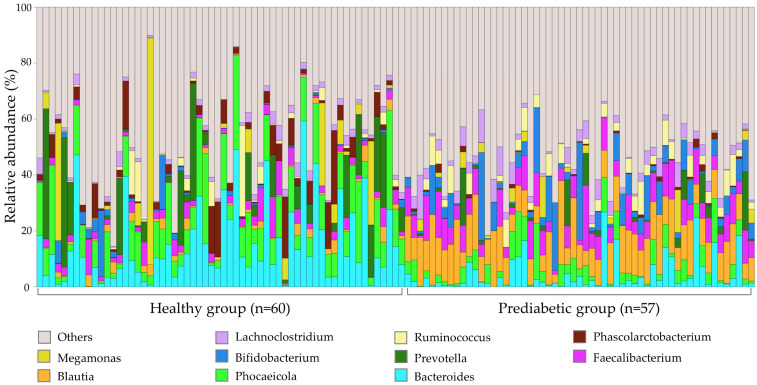
Gut microbiome profile of 117 fecal samples at the genus level. The remaining bacterial genera are summed as ‘Others’.

A total of 17,516,420 sequences were obtained. After further exclusion according to data completeness, 15,321,572 sequencing reads were used for further analyses (Supplementary Table S2). The top ten most abundant bacterial genera in all 117 samples are shown in different colors (Figure 1).

The LEfSe analysis results indicated 23 potential biomarkers in the healthy vs. prediabetes group and one biomarker in the carbohydrate group (Table 2). The average relative abundance of Bacteroides in healthy individuals’ fecal samples was higher than that in prediabetic individuals’ fecal samples. The average relative abundance of Blautia in prediabetic individuals’ fecal samples was higher than that in healthy individuals’ fecal samples.

**Figure 2:**
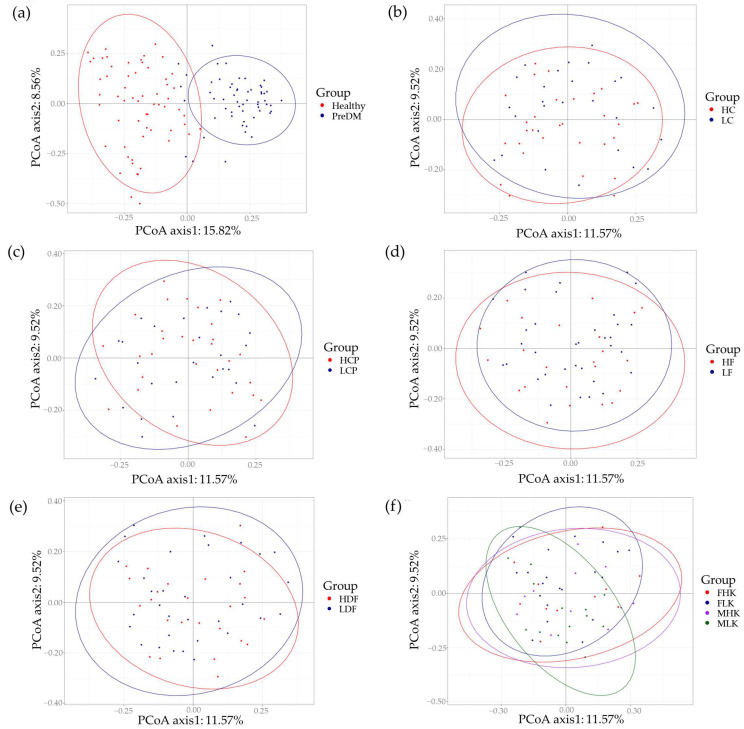
Visualization of beta diversity in different groups. (**a**) Healthy vs. prediabetes group. (**b**) Carbohydrate group. (**c**) Crude protein group. (**d**) Crude fat group. (**e**) Dietary fiber group. (**f**) Calories.

Principal coordinate analysis revealed that the microbiome profiles differed between the healthy and prediabetes groups (Figure 2a). However, the microbiome profiles were not different among the five prediabetes subgroups, which were grouped according to their nutrient intake, namely into carbohydrate (Figure 2b), crude protein (Figure 2c), crude fat (Figure 2d), dietary fiber (Figure 2e), and calorie groups (Figure 2f).

**Figure 3:**
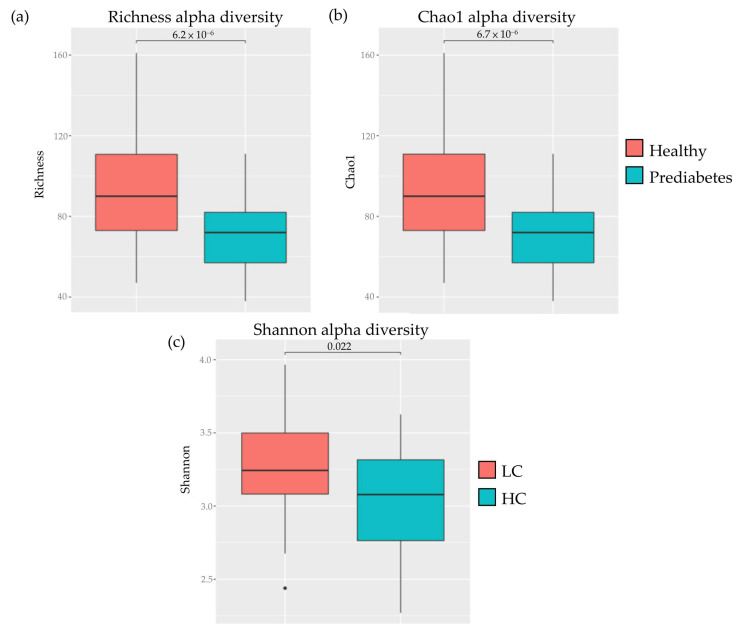
Alpha diversity of gut microbiome in healthy vs. prediabetes and carbohydrate groups. (**a**) Healthy vs. prediabetes (richness). (**b**) Healthy vs. prediabetes (Chao1 index). (**c**) Carbohydrate group (Shannon index).

The gut microbiome of the healthy group had a higher biodiversity than that of the prediabetes group (Figure 3a,b; Kruskal–Wallis test, *p* < 0.05). In the carbohydrate group, the gut microbiome of the LC group had a higher biodiversity than that of the HC group (Figure 3c; Kruskal–Wallis test, *p* < 0.05).

Overall, 9 and 14 bacterial species had higher average relative abundances in healthy and prediabetic fecal samples, respectively. The relative abundances of *Blautia*, *Faecalibacterium*, *Bifidobacterium*, *Clostridium*, *Anaerostipes*, *Mediterraneibacter*, and *Butyricicoccus* were higher in healthy fecal samples than in prediabetic fecal samples. *Streptococcus* and *Eggerthella* showed higher relative abundances in healthy fecal samples than in prediabetic fecal samples. *Bacteroides*, *Phascolarctobacterium*, *Parabacteroides*, and *Paraprevotella* were more abundant in prediabetic fecal samples.

In the carbohydrate group, the LC group showed a higher relative *Coprococcus* abundance in fecal samples than in the HC group’s fecal samples.

Table 3 shows the comparison of different functional digestive pathways between groups. The LC group had higher functional capabilities than the HC group, and the high crude protein (HCP) group had higher functional capabilities than the low crude protein (LCP) group for ‘fat digestion and absorption’ (ko04975). For fat metabolism, the functional capabilities of microbial communities were higher in healthy samples than in prediabetic samples for ‘fat digestion and absorption’ (ko04975), ‘fatty acid elongation’ (ko00062), and ‘synthesis and degradation of ketone bodies’ (ko00072). For blood glucose metabolism, healthy samples had higher functional capabilities than prediabetic samples in ‘glycosylphosphatidylinositol (GPI)-anchor biosynthesis’ (ko00563) and ‘glycosphingolipid biosynthesis-ganglio series’ (ko00604). There was no significant difference between the LF and HF groups in regards to the macronutrient metabolism.

## 4. Discussion

Diabetes mellitus is a metabolic disorder that leads to several complications. An imbalance in the gut microbiota can reportedly lead to abnormal insulin signaling and chronic low-grade inflammation, which are major causes of type 2 diabetes [23,37]. Gut dysbiosis increases permeability, which in return increases the contact of bacterium organelles with the immune cells and induces a long-term inflammatory immune response when left untreated [45]. In our study, the diversity and richness of the gut microbiota were markedly reduced in patients with prediabetes, similar to those in patients with DM [39]. Moreover, the composition of the gut microbiota in patients with prediabetes was found to be different from that in healthy individuals. A previous study showed that transplanting gut bacteria from healthy individuals into patients with metabolic syndrome could reduce insulin resistance [38], indicating how changing the gut microbiota may help individuals showing signs of prediabetes and diabetic patients.

Similar to the results of previous studies [23,25,29,32,33,34,44], this study found that *Blautia*, *Faecalibacterium*, *Bifidobacterium*, *Clostridium*, *Anaerostipes*, *Mediterraneibacter,* and *Butyricicoccus* abundances were relatively higher in healthy fecal samples than in prediabetic fecal samples. *Anaerostipes* and *Faecalibacterium* can help maintain the integrity of the intestinal mucosa by producing butyrate. The integrity of the intestinal mucosa can prevent pathogenic bacteria from entering the blood and destroying pancreatic β-cells, benefitting blood glucose control [35]. A study involving mouse models showed that *Clostridium* induces regulatory T cells to the colon and allows lymphocytes to enter inflamed islet cells to regulate the inflammatory response, thereby inhibiting diabetic morbidity [40]. However, contrary to previous findings [26,35], this study found that in healthy fecal samples, *Streptococcus* and *Eggerthella* were relatively more abundant than in prediabetic fecal samples. *Bacteroides*, *Phascolarctobacterium*, *Parabacteroides,* and *Paraprevotella* were more abundant in prediabetic fecal samples.

Multiple physiological metabolic pathways are significantly affected in patients with prediabetes [27]. This phenomenon leads to abnormal metabolism of GPI, defects in insulin transmembrane signaling, and retinoic acid-inducible gene I (RIG-I) overexpression, causing the immune system to attack β cells [46], which is not conducive to blood glucose control. Excessive activation of Akt/mTOR promotes DNA damage, increases the content of arginine catabolic mobile elements, damages the skin barrier, and allows bacteria such as *Staphylococcus aureus* to enter the body. This phenomenon results in low-density neutrophils and an increase in neutrophil extracellular traps; thus, wounds are not easily healed [28,41]. The dysregulation of sphingolipid metabolism is associated with insulin resistance and neuronal apoptosis, which in turn contribute to diabetic neuropathy [42]. In patients with DM, multiple lipid metabolic pathways are affected by an unbalanced diet. The gut microbiota of patients with prediabetes gradually changes and many physiological metabolic pathways are affected. Therefore, effectively improving an imbalanced gut microbiota may be a strategy to prevent the onset of diabetes.

Previous studies have shown that 57% of the gut microbiota is determined by diet, whereas genetic variation only accounts for approximately 12% [30]. Therefore, diet is a key factor in regulating the diversity of gut bacteria [31,43]. Dietary fiber can also be used as an energy source for the gut microbiota. Intestinal bacteria react with fermentable dietary fibers to produce short-chain fatty acids. Thus, those patients with prediabetes who maintain a low carbohydrate intake and a high amount of dietary fiber (HC, 8.6 g fiber/g carbohydrate; LC, 9.9 g fiber/g carbohydrate) have improved intestinal barrier integrity. The low carbohydrate intake group had a higher relative abundance of *Coprococcus*. Importantly, a previous study similarly reported that dietary fiber enriches *Coprococcus* [36]. *Coprococcus* has been shown to contribute to the production of butyrate [47], explaining the role of *Coprococcus* and dietary fiber in maintaining the gut barrier’s integrity. It is important to note that though the findings of this study highlight the role of *Coprococcus* and its difference in the LC and HC groups; many other bacterial genera (*Bifidobacterium*, *Lactobacillus*, *Faecalibacterium*, *Akkermansia*, etc.) play an equally significant role in modulating the integrity of the gut barrier [48], and therefore *Coprococcus* should not be the sole marker when evaluating gut integrity.

Microbial communities are related to several functional pathways (Table 3). When comparing LC with HC (LC, 22.6% total calories; HC, 35.4% total calories), the increased proportion of fat intake in the LC group resulted in the increased abundance of fat digestion and absorption pathways in this group than in the HC group. For Shiga toxin metabolism, HCP samples had higher functional capabilities than LCP samples in ‘pathogenic *Escherichia coli* infection’ (ko05130) and ‘shigellosis’ (ko05131).

Our study has several limitations. This study only recruited a small sample of prediabetic patients in Taiwan, and therefore the findings may not fully reflect the whole population. Furthermore, the individuals in the control group were not sampled from those living under the same environment or with a similar BMI. Socioeconomic and geographical factors and obesity have a direct correlation to the gut microbiome. The dietary composition of the two groups was not analyzed for differences, which may play a role in causing the difference in the gut microbiota observed in this study. Studies in the future should consider socioeconomic and geographical factors in their study design, and further studies with larger sample sizes should be performed to confirm the findings in this study.

## 5. Conclusions

Overall, we observed differences in the gut microbiota of prediabetic individuals compared to healthy individuals. This was reflected in the significantly different physiological and metabolic responses. A balanced intake of appropriate nutrients and a high-fiber diet may be helpful in maintaining normal physiological metabolism and diversity in the intestinal bacteria. Future studies should aim to understand the relationship of the gut microbiota and glycemic control in Asian populations.

## Figures and Tables

**Table 1:** Details of the fecal samples analyzed in this study.

Category	Male	Female	Total
Healthy	25	35	60
Prediabetes	26	31	57
Prediabetes group			
Carbohydrate group			
LC	14	13	27
HC	12	18	30
Crude Protein group			
LCP	10	17	27
HCP	16	14	30
Crude Fat group			
LF	14	20	34
HF	12	11	33
Dietary Fiber group			
LDF	15	17	32
HDF	11	14	25
Calories group			
FHK	0	11	11
FLK	0	20	20
MHK	11	0	11
MLK	15	0	15

**Table 2:** Biomarkers in healthy vs. prediabetes and carbohydrate groups.

Healthy vs. Prediabetes Group					
Healthy					
Bacterial genera	Group	LDA score	*p*-value	Fold change (healthy/prediabetes) *	Reference
*Bacteroides*	Healthy	4.70	6.92793 × 10^−8^	0.300	[25]
*Phocaeicola*	Healthy	4.55	8.30783 × 10^−8^	0.367	
*Alistipes*	Healthy	4.29	4.65658 × 10^−8^	0.074	
*Phascolarctobacterium*	Healthy	4.28	1.14771 × 10^−7^	0.121	[26]
*Parabacteroides*	Healthy	3.87	9.24002 × 10^−11^	0.190	[27]
*Paraprevotella*	Healthy	3.55	0.001328657	0.133	[28]
*Sutterella*	Healthy	3.25	1.29326 × 10^−5^	0.206	[29]
*Ruthenibacterium*	Healthy	3.19	2.69626 × 10^−9^	0.078	[30]
*Marseillibacter*	Healthy	3.01	6.86676 × 10^−8^	0.049	[31]
Prediabetes					
Bacterial genera	Group	LDA score	*p*-value	Fold change (healthy/prediabetes) *	Reference
*Blautia*	Prediabetes	4.75	2.94217 × 10^−20^	8.423	[29,32]
*Faecalibacterium*	Prediabetes	4.34	4.1797 × 10^−7^	2.335	[33,34]
*Bifidobacterium*	Prediabetes	4.32	3.62082 × 10^−8^	4.187	[35]
*Collinsella*	Prediabetes	4.22	1.76318 × 10^−7^	8.219	
*Fusicatenibacter*	Prediabetes	4.19	3.71865 × 10^−10^	9.128	[36]
*Streptococcus*	Prediabetes	4.11	1.00677 × 10^−9^	11.946	[33,37]
*Clostridium*	Prediabetes	4.01	1.40866 × 10^−6^	5.096	[38]
*Lachnoclostridium*	Prediabetes	3.94	0.001795935	1.985	
*Anaerostipes*	Prediabetes	3.92	3.32433 × 10^−9^	3.972	[39]
*Mediterraneibacter*	Prediabetes	3.89	2.233 × 10^−7^	5.735	[40]
*Butyricicoccus*	Prediabetes	3.57	4.19266 × 10^−8^	3.015	[41]
*Dorea*	Prediabetes	3.49	4.89717 × 10^−11^	6.086	[37,42]
*Clostridioides*	Prediabetes	3.18	8.49243 × 10^−6^	7.105	
*Eggerthella*	Prediabetes	3.02	1.11996 × 10^−8^	9.801	[43]
Carbohydrate group					
Bacterial genera	Group	LDA score	*p*-value	Fold change (HC/LC) *	
*Coprococcus*	LC	3.73	0.00920058	0.414	[44]

* Relative abundance of bacteria was used to calculate fold change.

**Table 3:** Differences in functional capabilities of microbial communities.

Group	Kegg ID	Kegg Pathway Name	Healthy/Prediabetes *	*p*-Value
healthy/prediabetes	ko04975	Fat digestion and absorption	6.36	6.312 × 10^−5^
	ko00062	Fatty acid elongation	0.17	4.219 × 10^−14^
	ko00563	GPI-anchor biosynthesis	64.97	1.528 × 10^−2^
	ko00604	Glycosphingolipid biosynthesis—ganglio series	2.04	9.552 × 10^−10^
	ko00072	Synthesis and degradation of ketone bodies	0.45	7.304 × 10^−12^
	Kegg ID	Kegg pathway name	HC/LC *	*p*-value
HC/LC	ko04975	Fat digestion and absorption	0.39	2.033 × 10^−2^
	Kegg ID	Kegg pathway name	HCP/LCP *	*p*-value
HCP/LCP	ko04975	Fat digestion and absorption	2.31	4.899 × 10^−2^

* Functional capabilities of microbial communities were used to calculate fold change.

## Data Availability

The datasets generated during and/or analyzed during the current study are available from the corresponding author on reasonable request due to the lack of public access of whole-sequencing data at http://www.ncbi.nlm.nih.gov/bioproject/891488 until 16 October 2026.

## Supplementary Materials

The following supporting information can be downloaded at: https://www.mdpi.com/article/10.3390/nu16081105/s1, Table S1. Nutrition intake of patients with prediabetes in 24 h, Table S2. Results of nucleic acid sequencing of 117 fecal samples.
