# Supplementary material for: Gut Microbiota in Patients with Prediabetes
Source: Nutrients. 2024 Apr 9;16(8):1105. doi: 10.3390/nu16081105 (PMC11053759; doi:10.3390/nu16081105)
Supplement: Supplementary file 1 [file nutrients-16-01105-s001.zip › nutrients-2939997-supplementary.pdf]

Supplementary Tables

Table S1 Nutrition intake of patients with prediabetes in 24 hours

	Gender	Calories (Kcal)	Carbohydrate (g)	Crude Protein (g)	Crude Fat (g)	Dietary Fiber (g)
Prediabetes1	Female	1066.43	220.28	36.69	7.73	18.86
Prediabetes2	Female	1430.55	285.22	30.66	24.96	13.05
Prediabetes3	Female	1056.17	183.87	48.19	20.31	19.91
Prediabetes4	Female	1754.17	249.65	86.99	49.79	21.64
Prediabetes5	Male	502.16	99.01	16.2	5.12	2.96
Prediabetes6	Male	1298.7	211.87	52.77	27.42	14.52
Prediabetes7	Female	1492.21	323.08	51.86	18.07	3.7
Prediabetes8	Male	2431.66	305.57	108.38	93.03	22.1
Prediabetes9	Male	1345.88	255.69	53.99	15.41	16.57
Prediabetes10	Male	1060.73	111.9	63.06	43.84	13.67
Prediabetes11	Female	821.83	145.11	38.21	13.29	16.96
Prediabetes12	Female	695.02	110.46	40.03	13.86	13.96
Prediabetes13	Male	3079.56	569.48	76.16	56.47	10.59
Prediabetes14	Male	1207.68	190.07	52.67	26.59	9.97
Prediabetes15	Female	2160.4	220.98	81.03	106.91	10.9
Prediabetes16	Female	1633.4	268.86	49.57	55.82	16.62
Prediabetes17	Male	2938.38	359.93	126.63	118.18	30.33
Prediabetes18	Male	1983.43	287.4	54.97	75.17	24.99
Prediabetes19	Female	986.43	122.71	50.16	35.51	9.21
Prediabetes20	Female	1487.54	216.06	34.56	56.32	11.89
Prediabetes21	Male	1943.03	301.77	93.46	50.78	37.03
Prediabetes22	Female	3027.58	318.41	132.06	144.06	37.72
Prediabetes23	Male	1836.21	289.35	75.67	47.44	31.49
Prediabetes24	Male	2253.34	298.12	103.57	77.61	25.78
Prediabetes25	Male	1450.5	98.79	60.49	93.75	12.41
Prediabetes26	Female	1321.55	212.02	45.93	35.87	3.93
Prediabetes27	Male	2505.27	220.4	141.45	123.61	11.22
Prediabetes28	Male	1863.08	279.59	66.31	57.19	9.52
Prediabetes29	Female	1216.56	168	43	43.11	11.22
Prediabetes30	Male	1103.5	149.6	34.55	49.13	25.35
Prediabetes31	Female	1863.08	279.59	66.31	57.19	9.52
Prediabetes32	Male	2112.51	311.15	63.19	72.01	14.02
Prediabetes33	Female	2450.63	431.57	85.08	45.12	17.97
Prediabetes34	Male	1509.19	178.41	79.45	54.1	8.01
Prediabetes35	Female	2007.31	324.1	64.72	64.11	55.39
Prediabetes36	Female	617.31	73.87	47.04	17.12	9.45
Prediabetes37	Female	1193.08	131.66	45.62	57.93	10.64
Prediabetes38	Female	1659.84	322.35	46.45	23.29	20.95
Prediabetes39	Female	1931.9	229.96	57.49	87.33	7.48
Prediabetes40	Female	839.83	110.26	32.74	33.16	15.04
Prediabetes41	Female	1599.8	150.82	71.34	81.12	17.94
Prediabetes42	Female	395.25	41.58	16.01	20.37	6.94
Prediabetes43	Male	834.68	97.73	29.31	36.8	5.76
Prediabetes44	Female	1146.34	137.94	68.7	37.04	8.78
Prediabetes45	Female	1319.51	139.18	54.31	62.87	13.41
Prediabetes46	Male	1081.72	117.45	41.56	49.86	2.89
Prediabetes47	Female	1155.53	133.85	57.65	45.49	9.4
Prediabetes48	Female	1418.69	207.31	56.16	45.76	21.57
Prediabetes49	Male	1900.97	277.27	66.99	59.36	7
Prediabetes50	Male	1414.77	128.41	72.96	71.68	10.34
Prediabetes51	Female	351.13	72.41	8.63	3.17	0.66
Prediabetes52	Male	1491.46	182.4	66.07	61.54	25.13
Prediabetes53	Female	1538.98	212.86	68.57	59.56	45.18
Prediabetes54	Male	1257.23	153.75	65.57	43.93	6.38
Prediabetes55	Male	1576.7	135.4	79.16	74.18	7.71
Prediabetes56	Male	583.51	109.57	20.94	6.45	1.39
Prediabetes57	Female	1050.76	128.66	68.67	37.66	29.78

Table S2 Results of nucleic acid sequencing of 117 fecal samples

Sample name	# of Raw reads	# of QC reads	QC%	Richness*	chao1*	Shannon*	Simpson*
Healthy1	143,332	131,092	91.46	93	93	3.4769	0.9515
Healthy2	84,458	74,761	88.52	83	83	2.7551	0.8238
Healthy3	80,207	71,554	89.21	52	52	2.7953	0.8943
Healthy4	103,015	93,582	90.84	68	68	2.4756	0.7966
Healthy5	84,448	71,946	85.2	92	93	2.7816	0.8113
Healthy6	150,757	126,764	84.08	114	114	3.4092	0.9199
Healthy7	132,384	114,387	86.41	77	78	3.0616	0.9268
Healthy8	99,623	89,034	89.37	74	74	3.2579	0.9347
Healthy9	124,101	108,738	87.62	100	100	3.1472	0.9267
Healthy10	221,690	207,366	93.54	97	97	3.1384	0.9263
Healthy11	140,764	128,747	91.46	80	81	2.6471	0.8764
Healthy12	261,188	231,642	88.69	115	115	3.5438	0.9470
Healthy13	207,542	188,806	90.97	118	118	3.3085	0.9320
Healthy14	171,377	153,452	89.54	104	104	3.2706	0.9135
Healthy15	122,531	113,310	92.47	63	63	2.6955	0.8986
Healthy16	166,637	143,173	85.92	116	116	3.4618	0.9360
Healthy17	220,077	190,210	86.43	113	113.5	2.8792	0.8806
Healthy18	135,983	121,999	89.72	93	93	3.2874	0.9351
Healthy19	191,272	168,043	87.86	47	47	0.9837	0.3386
Healthy20	147,699	113,184	76.63	67	67	2.5667	0.8212
Healthy21	175,163	160,306	91.52	64	64	2.5274	0.8443
Healthy22	135,980	124,844	91.81	138	138	3.6334	0.9514
Healthy23	178,857	157,261	87.93	92	92	3.0735	0.9216
Healthy24	104,922	97,093	92.54	110	110	3.2381	0.8996
Healthy25	207,617	178,902	86.17	142	142	3.8616	0.9646
Healthy26	144,823	127,015	87.7	71	71.5	2.2779	0.7895
Healthy27	160,661	136,066	84.69	77	77	2.8937	0.9079
Healthy28	156,257	126,929	81.23	90	90	3.1851	0.9336
Healthy29	136,261	115,851	85.02	104	104	3.1989	0.9268
Healthy30	276,427	244,511	88.45	68	68	2.4423	0.8444
Healthy31	162,122	133,784	82.52	73	73	2.7247	0.8766
Healthy32	165,391	148,198	89.6	87	87	3.3418	0.9454
Healthy33	151,452	130,810	86.37	72	72	2.6045	0.8694
Healthy34	87,773	81,234	92.55	96	96	2.9143	0.8693
Healthy35	228,268	179,295	78.55	102	102	3.1097	0.9156
Healthy36	192,432	170,330	88.51	161	161	4.1271	0.9746
Healthy37	187,812	165,444	88.09	117	117	3.4155	0.9390
Healthy38	169,964	136,557	80.34	55	55	2.7181	0.9052
Healthy39	181,194	145,323	80.2	89	89	3.1041	0.9258
Healthy40	154,297	126,016	81.67	85	85	2.9381	0.9049
Healthy41	212,154	171,638	80.9	73	73	1.7416	0.6699
Healthy42	182,113	147,677	81.09	94	94	3.3591	0.9405
Healthy43	188,200	167,557	89.03	121	121	3.8036	0.9675
Healthy44	150,273	126,789	84.37	67	67	2.5977	0.8582
Healthy45	208,561	166,949	80.05	114	114	3.7366	0.9627
Healthy46	192,242	170,937	88.92	91	91	3.0315	0.8958
Healthy47	179,324	159,621	89.01	80	80	2.7781	0.8780
Healthy48	172,310	152,148	88.3	62	62	2.3584	0.8404
Healthy49	178,226	160,353	89.97	109	109	3.0580	0.9033
Healthy50	172,741	152,656	88.37	84	84	3.0289	0.9197
Healthy51	164,173	142,883	87.03	122	122	3.5321	0.9299
Healthy52	202,407	178,778	88.33	90	90	3.3087	0.9400
Healthy53	194,068	178,933	92.2	120	120	3.0757	0.9023
Healthy54	235,597	198,654	84.32	125	125	3.3756	0.9298
Healthy55	176,923	158,584	89.63	63	63	2.0556	0.7993

Healthy56	159,974	145,183	90.75	83	83	2.9239	0.8912
Healthy57	177,011	160,779	90.83	89	89	3.0402	0.9018
Healthy58	185,740	153,935	82.88	75	75	2.7175	0.8471
Healthy59	134,358	113,495	84.47	131	131	3.7223	0.9550
Healthy60	88,734	72,202	81.37	66	66	2.7255	0.8455
Prediabetes1	107,329	93,417	87.04	54	54	2.7442	0.8984
Prediabetes2	73,078	55,526	75.98	65	65	3.0590	0.9168
Prediabetes3	186,025	153,591	82.56	67	67.5	2.8864	0.9241
Prediabetes4	115,824	98,190	84.78	77	77	3.0391	0.9080
Prediabetes5	112,431	94,603	84.14	51	52	2.2704	0.8373
Prediabetes6	126,646	108,781	85.89	85	85	3.5018	0.9470
Prediabetes7	131,859	101,077	76.66	88	88	3.2388	0.9323
Prediabetes8	94,953	79,654	83.89	73	73	3.2453	0.9392
Prediabetes9	128,196	102,170	79.7	48	48	2.6200	0.9008
Prediabetes10	98,761	81,968	83	76	76	3.1837	0.9257
Prediabetes11	101,182	85,595	84.6	86	86	3.2440	0.9375
Prediabetes12	152,776	128,493	84.11	78	78	3.3692	0.9482
Prediabetes13	136,894	121,165	88.51	49	49	2.6135	0.8824
Prediabetes14	140,782	118,032	83.84	52	52	2.5695	0.8611
Prediabetes15	151,063	117,000	77.45	60	60	2.8990	0.9176
Prediabetes16	142,188	115,165	80.99	77	77	3.3244	0.9428
Prediabetes17	128,981	105,759	82	96	96	3.2343	0.9334
Prediabetes18	182,983	143,002	78.15	82	82	3.3456	0.9417
Prediabetes19	59,024	50,911	86.25	87	87	3.5944	0.9556
Prediabetes20	179,229	155,856	86.96	66	66	3.1591	0.9380
Prediabetes21	158,503	134,493	84.85	100	100	3.3846	0.9444
Prediabetes22	205,581	168,926	82.17	57	57	2.6750	0.8935
Prediabetes23	199,562	171,112	85.74	72	72.5	3.2234	0.9390
Prediabetes24	164,300	155,756	94.8	67	67	3.0712	0.9239
Prediabetes25	80,620	67,805	84.1	46	46	2.4393	0.8608
Prediabetes26	73,235	61,763	84.34	46	46	2.8211	0.9130
Prediabetes27	61,682	53,606	86.91	97	97.5	3.5192	0.9444
Prediabetes28	75,700	66,247	87.51	51	51	2.5168	0.8563
Prediabetes29	165,381	143,671	86.87	64	64	2.6606	0.8726
Prediabetes30	98,786	86,665	87.73	74	74	2.9611	0.9007
Prediabetes31	89,584	83,773	93.51	108	108	3.5441	0.9505
Prediabetes32	68,706	59,448	86.53	78	78	3.2897	0.9410
Prediabetes33	110,080	99,163	90.08	94	100	3.0739	0.9161
Prediabetes34	178,295	162,356	91.06	105	106	3.4221	0.9528
Prediabetes35	59,984	56,768	94.64	42	42	2.9121	0.9280
Prediabetes36	65,608	62,234	94.86	67	68	3.0023	0.9297
Prediabetes37	58,956	45,310	76.85	71	71	3.2337	0.9391
Prediabetes38	144,154	136,449	94.66	56	56	3.0827	0.9182
Prediabetes39	70,506	57,840	82.04	79	79	3.4785	0.9523
Prediabetes40	193,799	180,989	93.39	70	71	3.1657	0.9348
Prediabetes41	169,371	158,303	93.47	81	81	3.6166	0.9600
Prediabetes42	92,172	85,225	92.46	44	44	3.0682	0.9369
Prediabetes43	197,153	182,597	92.62	93	93	3.7065	0.9602
Prediabetes44	175,178	161,233	92.04	81	81	3.4098	0.9437
Prediabetes45	142,621	132,068	92.6	58	58	3.2252	0.9414
Prediabetes46	197,680	180,795	91.46	69	69	3.2433	0.9431
Prediabetes47	135,452	123,944	91.5	56	56	3.0924	0.9364
Prediabetes48	181,896	169,569	93.22	86	86	3.5038	0.9525
Prediabetes49	216,982	200,081	92.21	88	88	3.6257	0.9612
Prediabetes50	190,994	180,194	94.35	72	72	3.4783	0.9551
Prediabetes51	176,678	166,147	94.04	57	57	3.2079	0.9413
Prediabetes52	165,244	155,327	94	111	111	3.9659	0.9689
Prediabetes53	125,208	117,403	93.77	38	38	2.5171	0.8840
Prediabetes54	128,129	120,317	93.9	63	63	3.2749	0.9465
Prediabetes55	132,784	123,722	93.18	80	80	3.6480	0.9616

Prediabetes56	173,197	161,758	93.4	69	69	2.9680	0.9042
Prediabetes57	140,568	131,250	93.37	74	74	3.6978	0.9657

***Alpha diversity of microbial communities**
